# Open-Source DNA-Encoded Library Package for Design, Decoding and Analysis: DELi

**DOI:** 10.1101/2025.02.25.640184

**Published:** 2025-03-01

**Authors:** James Wellnitz, Brandon Novy, Travis Maxfield, Ivanna Zhilinskaya, Shu-Hang Lin, Matthew Axtman, Tina Leisner, Jacqueline L. Norris-Drouin, Brian P. Hardy, Kenneth H. Pearce, Konstantin I. Popov

**Affiliations:** 1 Center for Integrative Chemical Biology and Drug Discovery, Chemical Biology and Medicinal Chemistry, Eshelman School of Pharmacy, University of North Carolina, Chapel Hill, North Carolina 27599, United States

## Abstract

DNA-encoded library (DEL) technology has become a powerful tool in modern drug discovery. Fully harnessing its potential requires the use of advanced computational methodologies, which are often available only through proprietary software. This limitation restricts flexibility and accessibility for academic researchers and small biotech companies, hindering the growth of the technology. Here, we present DELi, an open-source DEL informatics platform designed for library design, NGS decoding and calling, and enrichment analysis. To showcase its capabilities, we used DELi to design an in-house custom library (UNC-DEL006), a benzimidazole-based DEL, and performed proof-of-concept selection experiments against Bromodomain-containing Protein 4 (BRD4). The DELi decoding and analysis modules identified top-performing compounds, leading to the off-DNA synthesis of UNC 002–080, which was confirmed as a nanomolar BRD4 binder via isothermal titration calorimetry (ITC). In contrast, a chemically similar compound not prioritized by DELi, UNC 002–083, showed no measurable binding. These results demonstrate DELi as an effective tool for DEL design and analysis. Further, its open-source nature will promote ongoing development and contributions from the DEL community to expand its applications and capabilities.

## Graphical Abstract

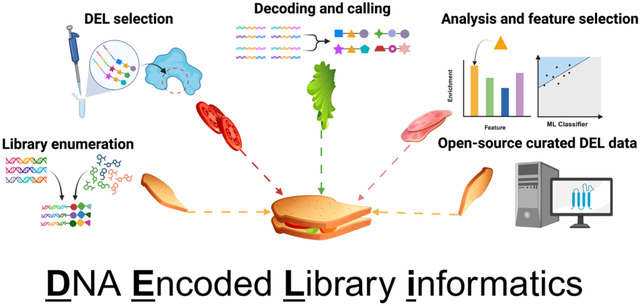

## Introduction

Drug discovery is a complex and costly process, with development often exceeding $1 billion and taking over a decade to bring a drug to market^1^. Technologies that enhance efficiency while reducing costs are essential to accelerating therapeutic development. High-throughput screening (HTS) has been a long-standing approach to speed up their stages of hit discovery, but its one-compound, one-well format is resource-intensive. To address this limitation, extensive work has gone into developing display technologies such as phage display and mRNA display to enable screening of millions to billions of compounds in a single tube^2^. However, these approaches are peptide-based and limited to natural and some unnatural amino acids^3^, limiting the diversity of flexibility of screening libraries. DNA-Encoded Libraries (DEL) has recently become a popular approach to still enable rapid screening of billion sized libraries, while allowing for the use of more drug like small molecules, significantly expanding chemical diversity for display technologies.

Since its inception in the 1990s^4^, DEL technology has advanced rapidly, leading to the discovery of potent and selective compounds for challenging targets such as GPCRs and epigenetic readers^5^. The commercialization of DELs by companies like WuXi, HitGen, and Charles River Laboratories has made screening billions to trillions sized DELs widely accessible^6^. However, while screening is streamlined, data analysis remains a major challenge. DEL selection results are complex, requiring expertise in chemistry and structure-activity relationships (SAR) to distinguish meaningful hits from artifacts. Despite the growing adoption of DELs, the lack of open-source computational tools for data analysis limits accessibility, creating barriers for researchers and small biotech companies looking to leverage the technology effectively.

To address this, we developed the DNA Encoded Library informatics (DELi) software package. DELi is a one-stop-shop for automated DEL-informatics pipeline development, with modules to support DEL design, full library enumeration, sequence demultiplexing and decoding, and automated selection analysis. DELi is an open-source academic initiative, with the goals of making recent advancements in DEL informatics easily available to the community with the hope to advance research by providing a solid foundation to build upon.

## Development & Modules

DELi is built using python and follows modern best practices for scientific software development. The package covers all aspects of DEL Informatics and is broken into several modules to provide an intuitive user experience. Some modules are designed to be accessed via a command line interface, while others are built to be utilized in custom scripts developed by users.

### Barcode Design

Error correcting DNA barcodes is a well-established method to help reduce the error rate of DNA sequencing^7^. Inclusion of such tags can recover as much as 10% of overall sequence reads^8^. Currently, DELi supports the design of hamming encoded DNA tags for single nucleotide polymorphism (SNP) correction through the design module. Standard parity hamming codes ensure a hamming distance of three between all barcodes, allowing correction of a single SNP. The module also supports an extra parity code, producing a hamming distance of four and enabling the detection, but not correction, of two SNP reads. Hamming encoded barcodes can range from a length of four to sixteen nucleotides. All valid barcodes will be generated for any given hamming code and can be reduced to remove any unwanted or undesired tags. Hamming encoded barcodes are meant to be applied to a specific region of the barcode, most commonly the building block regions, as they are most effective on small regions given the error rate of DNA sequencing.

### Library Enumeration

DELs are designed as a set of “building block” chemical fragments that can be joined together through a single reaction scheme. Libraries must be computationally enumerated to generate all the chemicals represented, which can sometime involve the creation of billions of compounds. DELi has built in support to do this enumeration from the basic information the user provides in the library information files (see Installation and Configuration). It supports both enumerating the entire library at once or enumerating single compounds on the fly based on their compound ID. A command line interface is also provided for this utility. Additional information can be found in the documentation.

### Barcode Decoding

**Figure 1:**
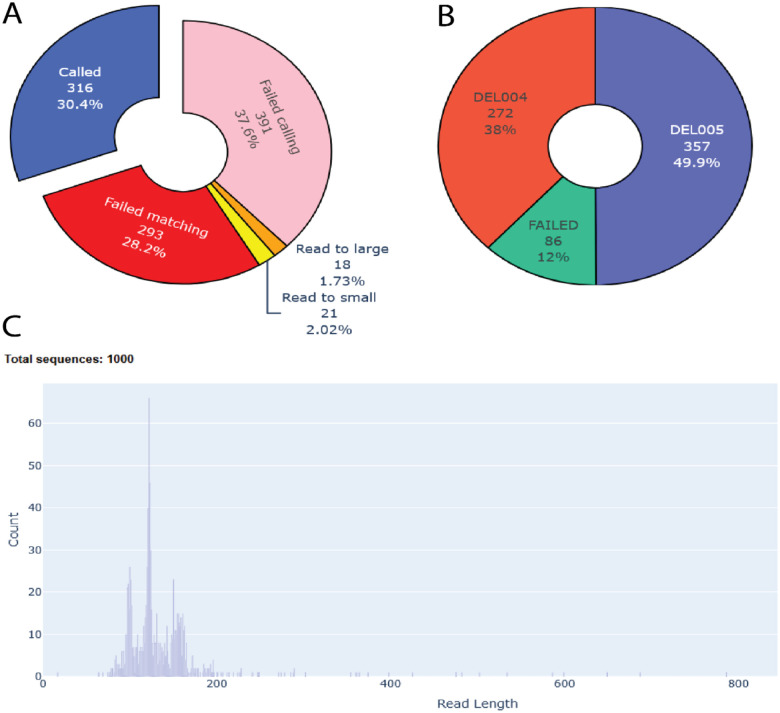
Example of some graphs generated by the DELi decoding HTML report: A) Pie chart showing how many reads failed to be demultiplex or called, as well as how many were successfully called. B) Pie chart of which libraries were found in the selection and at what percentages. C) Histogram of sequence read lengths read in from the fastq file.

A core component of the DEL informatics pipeline is converting the raw sequence reads collected post selection into compound counts for enrichment calculation. This is done by decoding the DNA sequences based on a lookup table of possible compounds. DELi support a quick and efficient process for such decoding. We utilize a semi-global alignment algorithm to anchor reads to a reference barcode with customizable error tolerance. After alignment, barcode sections can be mapped to the read allowing for decoding of individual sections. Decoding also supports unique molecule identifier (UMI) regions, allowing counts to be adjusted for uneven PCR amplification. DELi includes a robust decoding experiment initializer that is capable of handling large numbers of different DELs all with unique barcode set ups during decoding. It also supports demultiplexing separate DEL selection if sequences are not provided in a demultiplexed format. DELi can successfully decode up to 80% of reads at a rate of one million per minute on a standard workstation. The raw output of decoded sequence can be saved or converted into the common place “cube” format that maps each unique compound, its building block ids, and observed count for each selection to a single row in a CSV file. After decoding is finished, a detailed log file and digestible decoding HTML report (Fig 1) are generated for the user to see the overall results of the decoding.

### DEL Analysis

After decoding is performed, the decoded DEL selection data can be used with the DELi Analysis module to model the data quality and select potential hit compounds. We employ a suite of analytical techniques that attempt to identify trends in the target-enriched synthons and fully enumerated compounds. One such method is the normalized sequence count (NSC)^10^, which is functionally analogous to RPKM/TPKM^15^ in the RNA-seq literature or sequencing depth-based normalization in ChIP-Seq/ATAC-Seq experiments. The NSC normalizes the reads for a given DEL member by the sampling depth for that experiment where c_i_ represents the observed count for a library member and SD is the sampling depth for a given target (Eq. 1). One benefit of the NSC is that it doesn’t require a separate control experiment or naïve sequencing run—thus effectively cutting the costs and accessibility to conduct DEL experiments.

(1) $${\text{NSC}}_{i}=\frac{c_{i}}{SD}$$

Using this formulation of NSC, we calculate the merged maximum-likelihood enrichment ratio as proposed by Hou et al^11^. with a smoothing factor to account for inherent variance in DEL sequencing counts. Here c_1_ and c_2_ represent counts for a given library member from selection and control experiments respectively, while n represents total sequencing counts for that selection.

(2) $$R_{\text{MLE}}=\frac{n_{2}}{n_{2}}\times \frac{c_{1}+\frac{3}{8}}{c_{2}+\frac{3}{8}}$$

While users can provide DEL data without replicate samples, we opted for the merged calculation of MLE to increase confidence and raise the overall sequencing floor^11,16^. The normalized Z-score implemented by Faver et al^12^. models DEL selection data using a binomial distribution, which describes the probability of observing a given compound (or synthon/disynthon) × times across n independent trials with replacement. Here p_o_ represents the observed probability, p_i_ is the expected probability, and c_i_ are control counts for the given library member.

(3) $${\text{z}}_{\text{n}}=\frac{{\text{p}}_{\text{o}}-{\text{p}}_{\text{i}}}{1.4286\times \text{median}\left(\left|{\text{c}}_{\text{i}}-\text{median}\left(\text{c}\right)\right|\right)}$$

DELi also implements HitGen’s PolyO score^8^ for disynthon/monosynthon feature selection. This approach establishes a baseline score based on sequencing depth and size of a given DEL, then calculates the fold-change from the established baseline to determine if a feature is enriched. In addition to the above metrics, DELi offers a variety of optional graphical visualizations to assist in feature selection, including tools for analyzing competition experiments and visual rendering of compounds for structure-based selections. Furthermore, DELi incorporates multiple automated data balancing functions to enhance the performance of our machine learning models, which include both classification and regression-based approaches. These features are designed to streamline model generation and ensure more accurate and robust predictions in drug discovery workflows.

### Parallelization

While DELi provides no native support for parallelization within the package, it is built to be embarrassingly parallel in use. This enables trivial parallelization of most computed intensive tasks via simple workflow scripts. As an example, we provide a NextFlow workflow script that enables DELi decoding parallelized on a HPC system. Leveraging external workflow parallelization allows DELi to be efficiently deployed on nearly all infrastructures setups with minor customization.

## Installation and Configuration

DELi is made available for install via python pip. It can also be installed from source using python poetry. Installation with automatically install and add register DELi command line programs.

Some functionality, like decoding and enumeration, requires users to generate configuration files outlining the setup and contents of their DEL. Detailed documentation is provided on how to generate these files, with examples provided. Users only need to provide info on the library and its building blocks.

## Discussion

### Importance of Rigorous and Reproducible DEL Analysis

DEL technology allows for quick screening of millions to billions of compounds at once against a target of interest and the resulting sequencing output can provide insight into whether binding interactions occurred with library members. Decoding the sequencing results, however, is not quite as simple and can be overwhelming with large amounts of data that comes from DEL selections. Depending on the library size and on-target conditions screened, there could be hundreds of thousands of compounds that come through sequencing and analysis of which compounds to choose for off-DNA re-synthesis and testing can vary from one target to the next. Standardized procedures are not discussed in DEL literature as there are nuances on how to analyze selection output such as if there are known and validated binding pockets on the target or if there are known binders present in the selections as competitors with the DEL compounds. Standardization can also be difficult to establish as nomenclature is not universally maintained and could lead to confusion among different groups. Considerations for analyzing DEL data include, but are not limited to, reproducibility of the data, overall coverage of the library in the selections, cross-comparison of target conditions that provide possible insights into the binding events during the selections, and chemistry knowledge that can identify structural trends and similarities among compounds.

Reproducibility, like any experiment, is the pinnacle for building confidence that the compounds in sequencing experienced true binding events with the target. Without reproducibility, the compound selection process may be reduced to choosing compounds based on singular data points. Also important is the even coverage of the library members in the selection process which reduces compound bias and can build confidence that the binding events are real. Selection conditions that include known and validated binders or inhibitors, allow for cross-comparison with APO-target which can indicate binding events occurring in similar binding pockets. Additionally, selections without known binders or inhibitors but ones which include high and low protein quantities can create an environment where tighter binders can be pulled out compared to moderate to low binders. This pseudo-competition has been seen in literature as a strategy for targets without known inhibitors^17^. Without known binders or inhibitors included in the selection conditions, there is a higher threshold to overcome for identifying binders to desired binding pockets of the target. Since DEL selections are not immune to the pitfalls of screening which include promiscuous and allosteric binding, the more selection conditions included with any given target, the better the confidence can be in finding desired binders. Lastly, observing scaffold trends based on similar features of the compounds may be considered a traditional medicinal chemistry approach for selection analysis. With sequence counts of either two or three building block combinations as an aid to mark the abundance of any given compound present in sequencing, scaffold similarity analysis of structurally similar compounds allows for clustering the compounds together into ‘families’ containing those shared features. Triaging compounds into structurally similar families can aid in expediting the data analysis as similarly structured compounds may have similar activities with the target and thus can be grouped together. Representative members from each family can then be chosen for off-DNA testing based on sequence counts, structural features or other factors which are known to the target screened. Without triaging compounds into family groupings, the ability to keep track of and choose representative members for off-DNA synthesis and testing can become subjective. Sequencing counts can be used as a metric for choosing compounds but if similarly structured compounds are the highest enriched members, the representative pool for off-DNA synthesis can become monotoned.

### Open-science to Drive DEL and DEL-Machine Learning Advancement

Many large-scale DEL campaigns are carried out privately with limited access to the information gathered even after publication, hindering the potential for the community to reproduce and expand upon the work conducted. Commitment to sharing this data and the methodologies used to generate it is crucial to driving advancements in DEL. By open sourcing our entire analysis and DEL design pipeline, along with our DELs, we aim to address the limited availability of open-source DEL software and datasets. This in turn will lay a foundation for others to build upon, both by using standardized tools and enabling easier data processing DEL design.

This impact is not limited to just traditional DEL, but also in machine learning (ML) research efforts. Recent literature has discussed the beginning to investigate how to utilize DEL for ML^13,18–20^, and how to build ML tools that can assist all aspects of DEL. Yet, many groups that specialize in this type of research lack the ability to conduct DEL selection and create DELs in-house, hindering their ability to contribute to the field. By open sourcing our DEL, we hope to bridge that gap and enable these groups to apply and design new tools for DEL. Likewise, we will then be able to implement such advancements into DELi, enabling labs that specialize in DEL research but not ML to more easily apply the most recent advancements in computational approaches for DEL.

### Robust DEL Analysis is Important for DEL-ML

The rapid adoption of machine learning (ML) techniques in computational chemistry and drug discovery highlights the urgent need for better validation and standardization of data processing practices. Numerous studies have demonstrated that consistent errors or mis-annotations in chemical databases can significantly impair model performance, leading to inaccurate predictions^21,22^. These inaccuracies are not only detrimental at the model development stage but often propagate throughout the virtual screening process, compromising the reliability and efficiency of drug discovery workflows. Moreover, the shift towards open science necessitates the use of standardized ontologies, annotations, or dictionaries, alongside consistent analysis methods, to enhance data quality and quantity^23^. These efforts enhance the representation and understanding of chemical space across diverse regions, leading to more reliable and reproducible outcomes in computational chemistry and drug discovery. Machine learning models in drug discovery are most effective when trained and validated on data that is systematically curated and consistently annotated^24^. To address this, many initiatives, such as open databases like Open Targets^25^, are working to standardize data practices and provide high-quality, well-curated datasets that support more effective and accurate ML applications. In this context, we introduce the DELi platform, designed to address challenges related to enumeration, decoding, and normalization across DEL platforms, catering to both small academic laboratories and large pharmaceutical companies.

### BRD4 Case Study, DEL6

**Figure 2.**
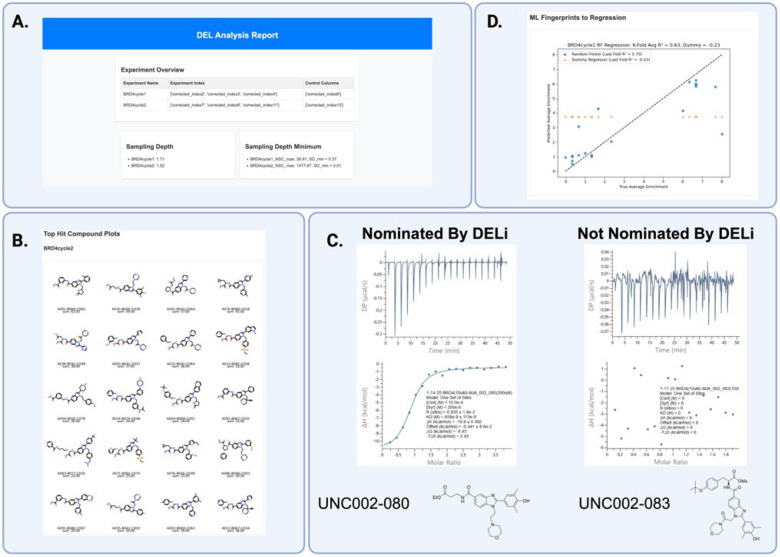
Automated DELi Analysis Report Prioritized nM Binder From DEL Selection. A) Header from DELi report detailing sampling depth and experimental information. B) Top trisynthon compounds for SAR analysis. C). ITC data for top nominated UNC002–080 showing nM binding activity compared to UNC002–083 which was structurally similar but not nominated by DELi’s automated report and showed no binding activity. D) Automated DEL-ML regression model created by DELi’s data balancing functions overlayed with dummy regressor to display overall results from 5-fold training regime.

To evaluate our DEL informatics pipeline, we employed DELi to design UNC-DEL006, a benzimidazole-based DNA-encoded library. Using DELi’s library enumeration module, we generated chemical structures, predicted physicochemical properties for the entire UNC-DEL006 library, and designed Hamming-encoded barcodes for the three-cycle building blocks. To validate our analytical workflow, we conducted selection experiments against the protein target Bromodomain-containing Protein 4 (BRD4), a well-studied protein target linked to cancer^26^. Through enrichment analysis, we identified top-performing molecular features and performed disynthon-based aggregation. From the prioritized compounds automatically reported in the DEL Analysis Report, we selected candidates for off-DNA synthesis and follow-up characterization. Notably, UNC #002–080 was confirmed as a nanomolar binder of BRD4 via isothermal titration calorimetry (ITC). In contrast, a structurally similar compound with a different disynthon feature, UNC #002–083—which was not prioritized by DELi—exhibited no detectable binding affinity by ITC (Figure 2C).

### Future Features

DELi has a detailed roadmap outlining new features and modules to be added in future updates. Prioritized updates include: improved generalizability of DEL configure to account for more complex library designs; built in machine learning options for DEL-ML virtual screening follow-up; improved command line interface; improved containerization and default workflows. As an open-source package, DELi accepts community feature requests as well as contributions following the contribution documentation.

## Conclusion

The field of DEL has rapidly expanded in recent years, with a surge in studies reporting novel DEL libraries, screening targets, and selection strategies^27^. Ready-to-purchase DELs have become increasingly available to academic labs and small biotech companies seeking to integrate this powerful technology into their drug discovery efforts^28,29^. However, many of these libraries require proprietary software licenses that limit flexibility and customization, leaving researchers constrained by closed systems. To address this, we introduce DELi, an open-source platform with fully accessible code and pipelines, available on GitHub for implementation and collaboration. Our goal is to provide researchers with a transparent and adaptable toolset, enabling greater control over their DEL workflows. We welcome feedback from the computational community and are committed to expanding DELi’s capabilities, including the expansion of deep learning models to explore novel, non-DEL-like chemical spaces for drug discovery.

## Figures and Tables

**Table 1.** DEL Statistical Methods Available in DELi

Method	References
NGS Sampling Depth	McCarthy et al. (2020)^9^
Normalized Sequence Count	Franzini et al. (2015)^10^
Maximum-Likelihood Enrichment Ratio	Hou et al. (2023)^11^
Normalized Z-Score	Faver et al. (2019)^12^
PolyO	Chen et al. (2022)^8^
DEL-Based Random Forest	McCloskey et al. (2020)^13^
DEL-Based Graph Convolutional Network and Graph Attention Network	Duvenaud et al. (2015)^14^
