# Supplementary material for: Open-Source DNA-Encoded Library informatics Package for Design, Decoding, and Analysis: DELi
Source: bioRxiv. 2025 Sep 17:2025.02.25.640184. Originally published 2025 Mar 1. Preprint. [Version 3] doi: 10.1101/2025.02.25.640184 (PMC11888370; doi:10.1101/2025.02.25.640184)
Supplement: 1 [file NIHPP2025.02.25.640184v3-supplement-1.pdf]

bioRxiv preprint doi: https://doi.org/10.1101/2025.02.25.640184; this version posted September 17, 2025. The copyright holder for this preprint (which was not certified by peer review) is the author/funder, who has granted bioRxiv a license to display the preprint in perpetuity. It is made available under aCC-BY 4.0 International license.

Figure S1. UNC DEL006 Synthesis.

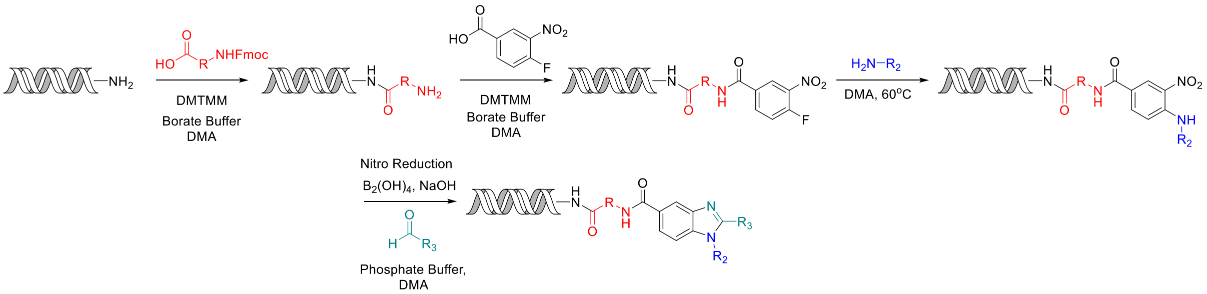

bioRxiv preprint doi: https://doi.org/10.1101/2025.02.25.640184; this version posted September 17, 2025. The copyright holder for this preprint (which was not certified by peer review) is the author/funder, who has granted bioRxiv a license to display the preprint in perpetuity. It is made available under aCC-BY 4.0 International license.

S1. DEL Synthesis Procedure

General Procedure of Acylation of HP

To a 1mM (2000 uL) solution of HP in 250 mM Borate Buffer (pH9.5) was added 40 equivalents of acid (440 uL of 200mM solution, 49.2 mg into 504 uL of DMA) followed by 35 equivalents of DMT-MM (390 uL of 200mM solution, 29.4 mg into 531 uL of Borate Buffer). The reaction was allowed to proceed at room temperature with gentle mixing overnight. At the completion of the reaction, the DNA was crashed out by addition of 10% total volume of 5M NaCl (283 uL) followed by 3x total volume of cold EtOH (9339 uL). This was placed in a -80C freezer for approximately 30 minutes then centrifuged at 4C to give pellet.

General Procedure for Nucleophilic Substitution

Resuspend the pellet to a 1mM solution with Borate Buffer and transfer 5 nmol of HP to 96 well plate to which 160 equivalents of amine were added (2.0 uL of 400mM solution). This mixture was heated to 60C for 16 hours. The DNA was then crashed out as previously described.

General Procedure for Nitro Reduction

Resuspend the pellets to a 1mM solution in water and pool into a single reaction flask to which 500 equivalents of NaOH (43.2 uL of 5 M NaOH solution) was added followed by 1130 uL of EtOH and finally 150 equivalents of $B_2(OH)_4$ (648 uL of 100mM solution) was added and allowed to react for 2 hours. The DNA was then crashed out a previously described, twice.

General Procedure for Benzimidazole formation

Resuspend the pellets to a 1mM solution in Phosphate Buffer (250 mM, Ph 5.5) was aliquoted back into a 96 well plate to which 60 equivalents of aldehyde (1.5uL of a 200mM solution in Acetonitrile) was added. This was allowed to react overnight. The DNA was then crashed out as previously described.

Ligation of DNA Barcodes

To a 1145uM solution of AOP-HP (19 uL in water) was split into 96 wells (150mM final concentration). 50.4 uL of a 500 uM Building block tag was added to each well (180 uM final concentration) followed by 10x T4 Ligase Buffer (14.0 uL), Water (53.8 uL) and finally T4 Ligase (8000 units/well, 2.8 uL). This was allowed to react at room temperature overnight. Ligations were confirmed by gel analysis and the DNA was crashed out as described above.

S2. Off-DNA Synthesis

bioRxiv preprint doi: https://doi.org/10.1101/2025.02.25.640184; this version posted September 17, 2025. The copyright holder for this preprint (which was not certified by peer review) is the author/funder, who has granted bioRxiv a license to display the preprint in perpetuity. It is made available under aCC-BY 4.0 International license.

ethyl 3-(4-fluoro-3-nitrobenzamido)propanoate (A). 4-fluoro-3-nitrobenzoic acid (104.2 mg, 1 Eq, 562.9 μmol) was dissolved in DMF (2.815 mL) to which ethyl 3-aminopropanoate (131.9 mg, 2 Eq, 1.126 mmol) was added. 2-(3H-[1,2,3]triazolo[4,5-b]pyridin-3-yl)-1,1,3,3-tetramethylisouronium hexafluorophosphate(V) (428.1 mg, 2 Eq, 1.126 mmol) was then added to the stirring solution and finally N-ethyl-N-isopropylpropan-2-amine (291.0 mg, 392 μL, 4 Eq, 2.252 mmol) was added dropwise and allowed to react overnight. The reaction was quenched by addition of water, extracted 3x with EtOAc, organics were washed with brine 2x, and solvents were removed to give crude material. TLC and LCMS show good conversion to desired product, so it was purified via normal phase chromatography using EtOAc/Hexanes to give desired product (86% yield).

ethyl 3-(4-((2-morpholinoethyl)amino)-3-nitrobenzamido)propanoate (B). Ethyl 3-(4-fluoro-3-nitrobenzamido)propanoate (46.0 mg, 1 Eq, 162 μmol) was dissolved in DMF (809 μL) to which 2-morpholinoethan-1-amine (42.1 mg, 2 Eq, 324 μmol)was added slowly. This reaction was then heated to 60 °C and allowed to react overnight. The reaction was quenched by addition of water, extracted 3x with EtOAc, organics were washed with brine and solvents were removed to give crude material. TLC and LCMS show complete conversion of starting material to desired product, but the LCMS shows an additional more polar peak that could correspond to the amine. The reaction was purified via normal phase chromatography (DCM/MeOH) to give the desired product (73% yield).

ethyl 3-(2-(4-hydroxy-3,5-dimethylphenyl)-1-(2-morpholinoethyl)-1H-benzo[d]imidazole-5-carboxamido)propanoate (UNC11951). Ethyl 3-(3-amino-4-((2-morpholinoethyl)amino)benzamido)propanoate (43.0 mg, 1 Eq, 118 μmol) was dissolved in DMF (787 μL) to which 4-hydroxy-3,5-dimethylbenzaldehyde (35.4 mg, 2 Eq, 236 μmol) was heated to 60 °C overnight. The material was quenched by addition of water, extracted 3x with EtOAc, combined organics were washed with brine, dried with sodium sulfate and the solvents were removed to give crude material. It was purified via reverse phase chromatography to give desired product (68% yield).

S3. Analysis Background

We employ a suite of analytical techniques that attempt to identify trends in the target-enriched synthons and fully enumerated compounds. One such method is the normalized sequence count (NSC)[32], which is functionally analogous to RPKM/TPKM[35] in the RNA-seq literature or sequencing depth-based normalization in ChIP-Seq/ATAC-Seq experiments. The NSC normalizes the reads for a given DEL member by the sampling depth for that experiment where c_i represents the observed count for a library member and SD is the

bioRxiv preprint doi: https://doi.org/10.1101/2025.02.25.640184; this version posted September 17, 2025. The copyright holder for this preprint (which was not certified by peer review) is the author/funder, who has granted bioRxiv a license to display the preprint in perpetuity. It is made available under aCC-BY 4.0 International license.

sampling depth for a given target (Eq. 1). One benefit of the NSC is that it doesn't require a separate control experiment or naïve sequencing run—thus effectively cutting the costs and accessibility to conduct DEL experiments.

$$\mathrm{NSC}_i = \frac{c_i}{SD} \tag{1}$$

Using this formulation of NSC, we calculate the merged maximum-likelihood enrichment ratio as proposed by Hou et al[33]. with a smoothing factor to account for inherent variance in DEL sequencing counts. Here c_1 and c_2 represent counts for a given library member from selection and control experiments respectively, while n represents total sequencing counts for that selection.

$$R_{\mathrm{MLE}} = \frac{n_2}{n_1} \times \frac{c_1 + \frac{3}{8}}{c_2 + \frac{3}{8}} \tag{2}$$

While users can provide DEL data without replicate samples, we opted for the merged calculation of the MLE ratio to increase confidence and raise the overall sequencing floor[33,36]. The normalized Z-score implemented by Faver et al[21]. models DEL selection data using a binomial distribution, which describes the probability of observing a given compound (or synthon/disynthon) x times across n independent trials with replacement. Here p_o represents the observed probability, p_i is the expected probability, and c_i are control counts for the given library member.

$$\mathrm{z_n} = \frac{\mathrm{p_o} - \mathrm{p_i}}{1.4286 \times \mathrm{median}(|\mathrm{c_i} - \mathrm{median}(\mathrm{c})|)} \tag{3}$$

DELi also implements HitGen's PolyO score[23] for disynthon/monosynthon feature selection. This approach establishes a baseline score based on sequencing depth and size of a given DEL, then calculates the fold-change from the established baseline to determine if a feature is enriched.
